# PDGF Receptor Alpha+ Mesoderm Contributes to Endothelial and Hematopoietic Cells in Mice

**DOI:** 10.1002/dvdy.23923

**Published:** 2013-01-17

**Authors:** Guo Ding, Yosuke Tanaka, Misato Hayashi, Shin-Ichi Nishikawa, Hiroshi Kataoka

**Affiliations:** 1 Laboratory for Stem Cell Biology, RIKEN Center for Developmental BiologyKobe, Japan; 2 PRESTO, Japan Science and Technology AgencySaitama, Japan

**Keywords:** PDGFRα, Flk-1, Runx1, endothelial, hematopoiesis

## Abstract

Background: Early mesoderm can be classified into Flk-1+ or PDGF receptor alpha (PDGFRα)+ population, grossly representing lateral and paraxial mesoderm, respectively. It has been demonstrated that all endothelial (EC) and hematopoietic (HPC) cells are derived from Flk-1+ cells. Although PDGFRα+ cells give rise to ECs/HPCs in in vitro ES differentiation, whether PDGFRα+ population can become hemato-endothelial lineages has not been proved in mouse embryos. Results: Using PDGFRαMerCreMer mice, PDGFRα+ early mesoderm was shown to contribute to endothelial cells including hemogenic ECs, fetal liver B lymphocytes, and Lin-Kit+Sca-1+ (KSL) cells. Contribution of PDGFRα+ mesoderm into ECs and HPCs was limited until E8.5, indicating that PDGFRα+/Flk-1+ population that exists until E8.5 may be the source for hemato-endothelial lineages from PDGFRα+ population. The functional significance of PDGFRα+ mesoderm in vascular development and hematopoiesis was confirmed by genetic deletion of *Etv2* or restoration of *Runx1* in PDGFRα+ cells. *Etv2* deletion and *Runx1* restoration in PDGFRα+ cells resulted in abnormal vascular remodeling and rescue of fetal liver CD45+ and Lin-Kit+Sca-1+ (KSL) cells, respectively. Conclusions: Endothelial and hematopoietic cells can be derived from PDGFRα+ early mesoderm in mice. PDGFRα+ mesoderm is functionally significant in vascular development and hematopoiesis from phenotype analysis of genetically modified embryos. *Developmental Dynamics 242:254–268, 2013*. © 2013 Wiley Periodicals, Inc.

**Key Findings:**

- PDGF receptor alpha–positive mesoderm contributes to endothelial and hematopoietic cells in physiological mouse embryogenesis.
- PDGF receptor alpha–positive mesoderm in early embryo is distinct from yolk sac blood island mesoderm, representing a source of hematopoietic cells on the embryo proper side.
- Genetic manipulation of *Etv2* or *Runx1* in PDGF receptor alpha–positive mesoderm demonstrates the functional significance of this mesoderm subset in vascular development and hematopoiesis.

## INTRODUCTION

**Fig. 1:**
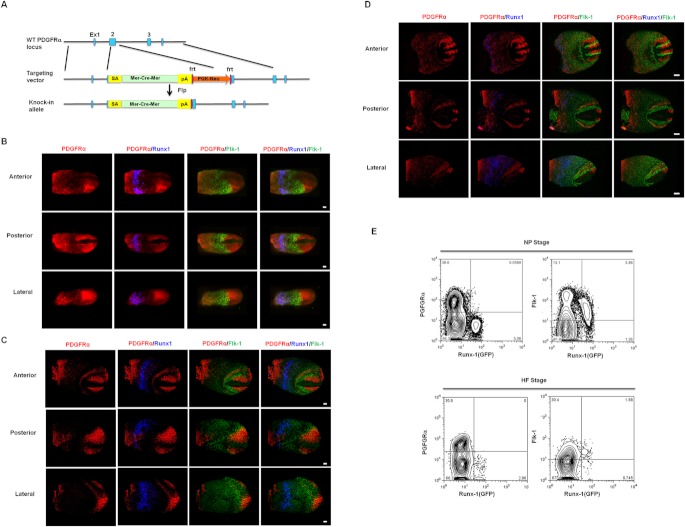
**A**: Generation of PDGFRα-MerCreMer (PRα-MCM) knock-in mice. Tmx-inducible MerCreMer was knocked into the PDGFRα locus using homology arms corresponding to 5′ side, 79,307–85,194; 3′ side, 85,253–89,284 from RP23–55P22. Correct integration was confirmed by Southern blotting. PCR genotyping can be performed by primers, PRα WT Rev1; ggagaacaaggacgcatgtgtgg, PRα5′F2; gcttccctcttatctatctgactg, MCM Rev2; aaggtggacctgatcatggagattc (WT, 260 bp; Targeted, 700 bp). SA, splice acceptor; pA, polyadenylation signal. **B:** Immunostaining of PDGFRα, Flk-1, and Runx1 in NP stage (E7.5) mouse embryos. NP-stage wild type embryos were stained by PDGFRα, Flk-1, and Runx1 antibodies. There was a narrow overlap of PDGFRα and Flk-1 domains. Note, however, that PDGFRα and Runx1 expression was seen in distinct mesoderm subsets. Scale bar=100 μm. **C:** Immunostaining of PDGFRα, Flk-1, and Runx1 in HF stage (E8.0) mouse embryos. In HF-stage embryos, similar staining was observed as in NP-stage samples. Note also in this stage that there is no overlap between PDGFRα and Runx1+ populations. Scale bar=100 μm. **D:** Immunostaining of PDGFRα, Flk-1, and Runx1 in E8.5 somite stage embryos. As seen in E7.5 or E8.0 embryos, there is some overlap between PDGFRα and Flk-1 areas, however no overlap between PDGFRα and Runx1. In this stage, PDGFRα or Flk-1 antibody stains form somite structure or aortas, respectively. Scale bar=100 μm. **E:** FACS analysis of NP- and HF-stage embryos. NP- (left) or HF- (right) stage embryos were stained by indicated antibodies to examine the relationship between Runx1, PDGFRα, and Flk-1. In both stages, almost no Runx1+/PDGFRα+ cells were detected, while more that 70% of Runx1-GFP+ cells were Flk-1+.

Mesoderm is an intermediate state for epiblasts or ES cells to develop into endothelial (EC) and hematopietic cells (HPC). Early mesoderm can be classified grossly into Flk-1+ lateral-extraembryonic or PDGFRα+ paraxial mesoderm. While Flk-1+ mesoderm has been shown to contribute to virtually all HPCs and ECs (Lugus et al., 2009), several reports indicate that HPCs and ECs originate from somite, intraembryonic mesenchyme, or allantois containing PDGFRα+ mesoderm (Jukkola et al., 2005; Bertrand et al., 2005; Zeigler et al., 2006; Corbel et al., 2007). Among candidate origins for HPCs and hematopoietic stem cells (HSCs), yolk sac (YS) (Kataoka et al., 1997; Yoder et al., 1997; Samokhvalov et al., 2007) and lateral mesoderm (Wasteson et al., 2008) consist mainly of Flk-1+ cells with few or not so many PDGFRα+ cells. In contrast, more PDGFRα+ cells are present in intraembryonic para-aortic splanchnopleura (p-Sp) (Cumano et al., 1996), placenta (Gekas et al., 2005), and allantois, raising the possibility that PDGFRα+ cells can generate HPCs from these sites (Takakura et al., 1997). It has been demonstrated that PDGFRα+ mesoderm generates ECs and HPCs in ES differentiation culture (Sakurai et al., 2006). However, in vitro culture system defines each cell population by marker staining but lacks anatomical information that is critical to understand the physiological differentiation process. In mouse embryos, we have demonstrated that Flk-1+/PDGFRα+ cells accumulate in the absence of *Etv2,* failing to differentiate into Flk-1+/PDGFRα-cells (Kataoka et al., 2011). This suggests that PDGFRα+ cells can contribute to ECs and HPCs in mouse embryogenesis. In mouse development, however, how PDGFRα+ population including Flk-1+/PDGFRα+ cells contribute to various cell types has not been thoroughly evaluated. It is also important to confirm if the differentiation pathway in in vitro ES cell differentiation can be recapitulated in the real animal. In ES differentiation, it is expected that PDGFRα+/Flk-1+ cells are multi-potential for hemato-endothelial, muscle, or mesenchymal lineages partly due to the greater plasticity of differentiating ES cells. Since Flk-1+ cells have been shown to differentiate into skeletal muscle and cardiomyocytes in mouse embryos (Motoike et al., 2003), it is possible that PDGFRα induction in Flk-1+ cells might enforce the differentiation of Flk-1+ cells preferentially into muscle or mesenchymal lineages in the in vivo context. Therefore, we examined if PDGFRα+ cells contribute to ECs and HPCs in mouse embryos where differentiation is controlled in a more physiological manner. For this purpose, PDGFRα-MerCreMer (PRα-MCM) knock-in mice, expressing tamoxifen (Tmx) inducible MerCreMer (MCM) under control of the PDGFRα locus (Fig. 1A), was crossed with ROSA26-LacZ or YFP reporter strains (PRα-MCM-LacZ or PRα-MCM-YFP mice) to trace labeled PDGFRα+ cells in mouse embryos. We focused on ECs and HPCs derived from PDGFRα+ cells, as this may help to clarify the origin of HSCs that are one of the most important cell types to be created for therapeutic purposes.

## RESULTS

### PDGFRα Mesoderm Is Distinct From Extraembryonic Runx1+ Mesoderm in Early Embryos

To locate the PDGFRα+ mesoderm, E7.5 neural plate (Fig. 1B), E8.0 head fold (Fig. 1C), or E8.5 somite stage (Fig. 1D), embryos were immunostained by PDGFRα, Flk-1, and Runx1 antibodies. As we reported, PDGFRα and Flk-1 stained almost distinct subset of mesoderm with some overlap in lateral mesoderm closer to the paraxial region (Kataoka et al., 1997, 2011). Runx1 was used to stain HPC precursors including erythroid progenitors and part of HSCs (Tanaka et al., 2012). No clear overlap was observed between PDGFRα+ and Runx1+ mesoderm, indicating that PDGFRα or Runx1 specifies distinct mesoderm population. This result was also confirmed by FACS analysis of NP- and HF-stage Runx1-Venus Knock-in embryos, in which almost no PDGFRα+/Runx1+ cells were detected (Fig. 1E). In situ hybridization for *Runx1* also revealed that its expression is limited in the proximal region of the extraembryonic yolk sac, namely the blood island, validating that our immunostaining by Runx1 antibody for multi-color detection correctly reflects *Runx1* in situ hybridization (data not shown). These findings suggest that any HPCs coming from PDGFRα+ cells develop from those cells that do not express Runx1 in these stages. At E7.5–8.5, we were able to detect an area stained by both PDGFRα and Flk-1. This double-positive population almost disappeared at E9.5 (see Fig. 6C), indicating that vasculogenic capacity in PDGFRα+ cells is dependent on Flk-1 and is limited in early time frame during embryogenesis.

### PDGFRα+ Cells Labeled at E7.5–8.0 Contribute to Endothelial Cells Including Aorta-Gonad-Mesonephron (AGM)

**Fig. 2:**
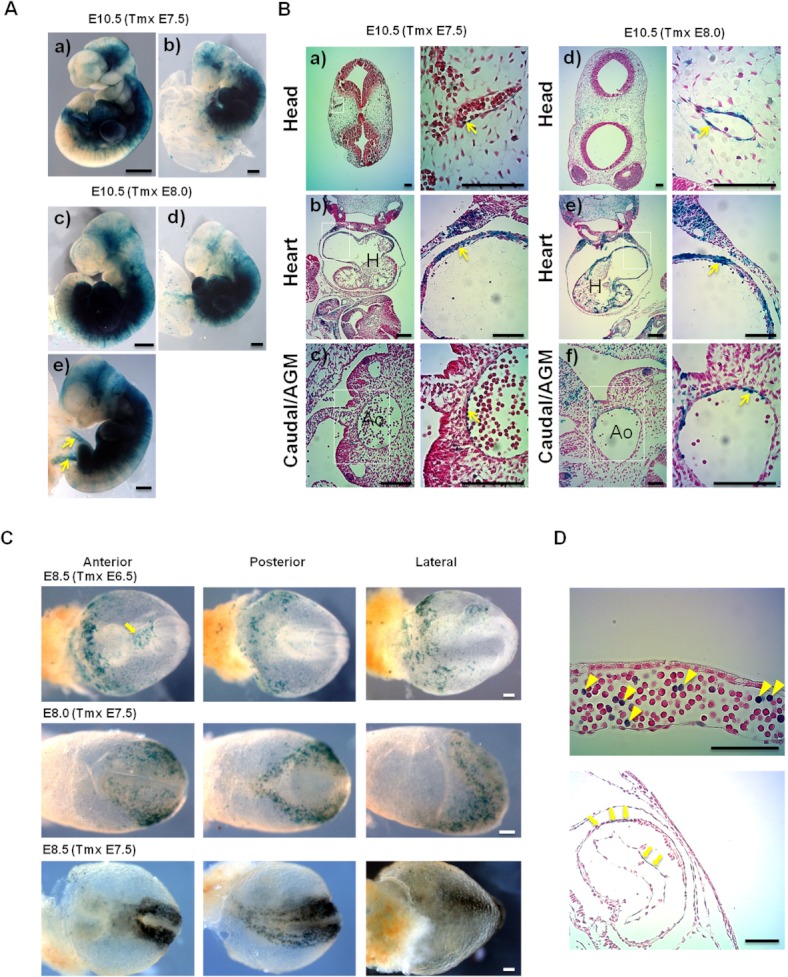
**A**: Whole mount LacZ staining of PRα-MCM-LacZ embryos. To activate MerCreMer, Tmx was injected into pregnant females at E7.5 (neural plate stage, top panels) or E8.0 (head fold stage, middle panels), when PDGFRα starts to be clearly detected in mesoderm with initiation of vasculogenesis and hematopoiesis. Embryos were LacZ stained at E10.5. LacZ-stained cells were distributed mainly in the intraembryonic area including the head, heart, and umbilical cord (arrows in lower panel). Note that only a few labeled cells were seen in the yolk sac except the umbilical cord (b, d). Scale bar=1 mm. **B:** Histological analysis of LacZ-stained PRα-MCM-LacZ embryos with Tmx injection at E7.5 (**a–c**) or E8.0 (**d–f**) into pregnant females. Embryos were LacZ stained and sectioned at E10.5. LacZ-positive cells were distributed in cranial mesenchyme, atrial and ventricular cardiomyocytes. Higher magnification is shown on the right side of a–f. Note the presence of labeled ECs in cranial vessels (a, d) and ventral wall of the descending aorta (arrows on right in c, f). Presence of LacZ+ ECs and HPCs in the aorta-gonad-mesonephron region is consistent with the finding that fetal liver HSCs exist as E7.5–8.5 labeled cells in the fetal liver (see Figs. 3A and 6A,a). Scale bar=100 μm. C, D: Short-term tracing of PDGFRα+ cells from E7.5 to E8.0–8.5. **C:** PRα-MCM-LacZ embryos were analyzed at E8.0 and E8.5 after Tmx injection into pregnant females at E6.5 or E7.5. Note that labeled cells are present in the lateral mesoderm area that overlaps Flk-1 staining (see Fig. 1C) at E8.0 (“Posterior” panels). Existence of some labeled cells in this lateral area where Flk-1+ cells exist at E8.0 supports that part of the labeled cells might become Flk-1+. At E8.5,labeled cells become more concentrated around the paraxial zone (bottom panels). Tmx injection at E6.5 labeled YS HPCs that are different from those labeled by E7.5 injection (top panels). Scale=100 μm. **D:** Sections of LacZ-stained E8.5 embryos after E6.5 injection demonstrate the labeling in YS blood islands (arrowheads) and cardiac tissue (arrows). Note that part of YS HPCs (top panel) and cardiac tissue (bottom panel) contains LacZ+ cells. Scale=100 μm.

To trace the fate of early mesoderm PDGFRα+ cells, pregnant females were injected with Tmx at E7.5 or E8.0 and PRα-MCM-LacZ embryos were analyzed at E10.5. In PRα-MCM-LacZ embryos, cells labeled at E7.5–E8.0 distributed broadly inside the embryo including somite, head mesenchyme, and heart at E10.5. LacZ+ cells mainly distributed throughout the embryo proper side with fewer labeled cells in YS, indicating that PDGFRα+ early mesoderm almost exclusively contribute to the embryo proper (Fig. 2A). Histological analysis of LacZ-stained embryos revealed that PDGFRα+ cells labeled at E7.5 or E8.0 contribute widely to somites, mesenchyme, cardiomyocytes, and ECs (Fig. 2B).

As we aimed to analyze the contribution of PDGFRα+ cells into ECs and HPCs, LacZ-stained embryos were examined if there were any ECs labeled, especially those regarded as hemogenic ECs. We found that some ECs were LacZ positive in PRα-MCM-LacZ embryos exposed to Tmx at E7.5–8.0 (Fig. 2B). Notably, ECs on the ventral side of aorta in the AGM region were LacZ positive, including HPCs budding from the EC layer (Fig. 2B). This finding demonstrates that PDGFRα+ cells labeled at E7.5–8.0 can contribute to hemogenic ECs that potentially give rise to definitive HPCs. Tmx injection at E6.5 failed to label PDGFRα+ mesoderm derivatives such as somites or paraxial mesoderm (Fig. 2C), which were extensively labeled by E7.5 injection. Rather, E6.5 Tmx injection labeled mainly extraembryonic part including YS HPCs, demonstrating the time frame specificity of labeling in PRα-MCM line (Fig. 2C), as 24 hr delay of Tmx injection resulted in the labeling of distinct populations. These findings suggest that PRα-MCM transgene has labeled in relevant cell types that are expected to be the descendants of PDGFRα+ cells including cardiomyocytes and head mesenchyme. Additionally, we observed LacZ-positive ECs including those with potential hemogenic capacity. Tmx injections at E6.5 and E7.5 also support our assumption that Tmx will be almost ineffective 24 hr after injection (Zovein et al., 2008), ensuring the time frame specificity of the PRα-MCM transgene.

### PDGFRα+ Cells Labeled at E7.5–8.0 Contribute to Fetal Liver HPCs and ECs at E15.5

**Fig. 3:**
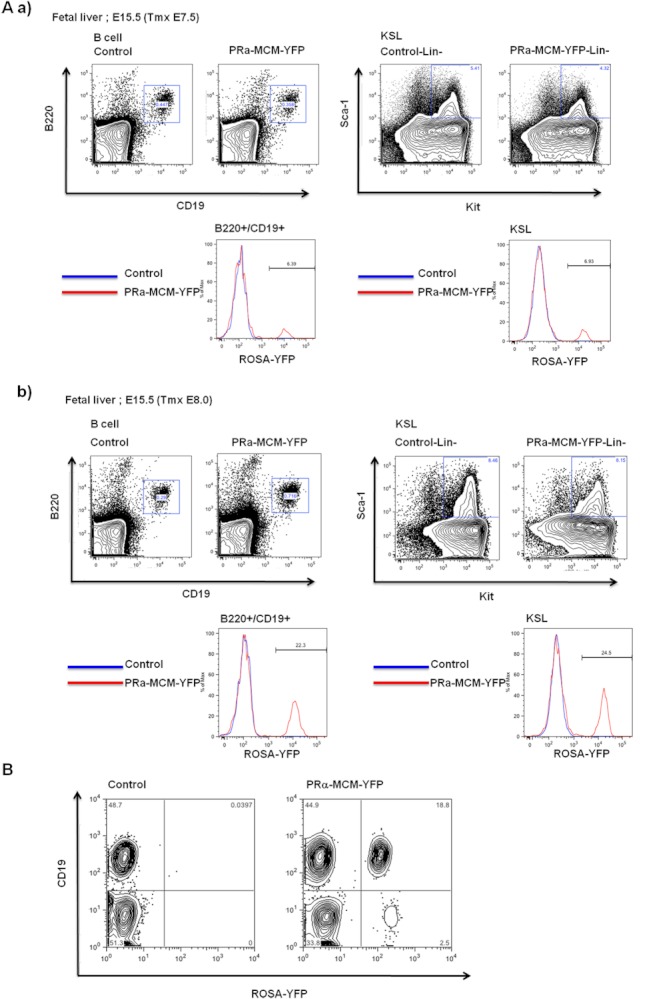
**A**: PDGFRα+ cells labeled at E7.5 or E8.0 contribute to fetal liver HPCs. **a:** Pregnant females with PRα-MCM-YFP embryos were Tmx injected at E7.5. Contribution of YFP+ cells to fetal liver HPCs was analyzed at E15.5. To evaluate the contribution of labeled cells to definitive HPCs, B cell and KSL populations were gated. YFP+ cells constituted 6–7% of B or KSL population gated. (YFP expression from ROSA locus is the indicator of Cre recombination by PRα-MCM transgene.) **b:** PRα-MCM-YFP embryos were analyzed at E15.5 after Tmx injection at E8.0 into pregnant females. To evaluate the contribution of labeled cells to definitive HPCs, B cell and KSL populations were gated. YFP+ cells constituted 22 or 25% of B and KSL population, respectively. **B:** PDGFRα+ cells from embryo proper develop into B cells in explant culture. After Tmx injection into pregnant females at E7.5, caudal part of E8.25 PRαMCM-YFP embryos was explanted and cultured on OP9 feeder cells with IL-7 (20 ng/ml) and Flt-3 ligand (10 ng/ml). After 2 days, explants were dissociated and further cultured in the same condition for another 2 weeks. Floating cells were harvested and stained by anti-CD19 antibody to demonstrate the B cell generation. Note that significant proportion of CD19+ B cells exist as YFP+ in cultured cells from the PRαMCM-YFP embryo (right panel).

To analyze the contribution of PDGFRα+ early mesoderm into HPCs, especially for fetal liver HPCs, ECs/HPCs in PRα-MCM-YFP embryos were analyzed by FACS at E15.5 after E7.5 or E8.0 Tmx injection into pregnant females. After Tmx injection at E7.5, 6–7% of B lymphocytes and KSL cells were YFP+, indicating the contribution of PDGFRα+ mesoderm to definitive type HPCs (Fig. 3A,a). Tmx injection at E8.0 labeled over 20% of B cells or KSL cells at E15.5 (Fig. 3A,b). Since most of PDGFRα+ cells reside in the intraembryonic area at E8.0, this finding is consistent with the report that intraembryonic caudal mesenchymal cells develop into aorta-gonad-mesonephron (AGM) HSCs (Bertrand et al., 2005). PDGFRα+/Etv2-YFP+ cells, potentially developing into ECs/HPCs, are also demonstrated to exist on the embryo proper side at E7.5 (Koyano-Nakagawa et al., 2012).

**Fig. 4:**
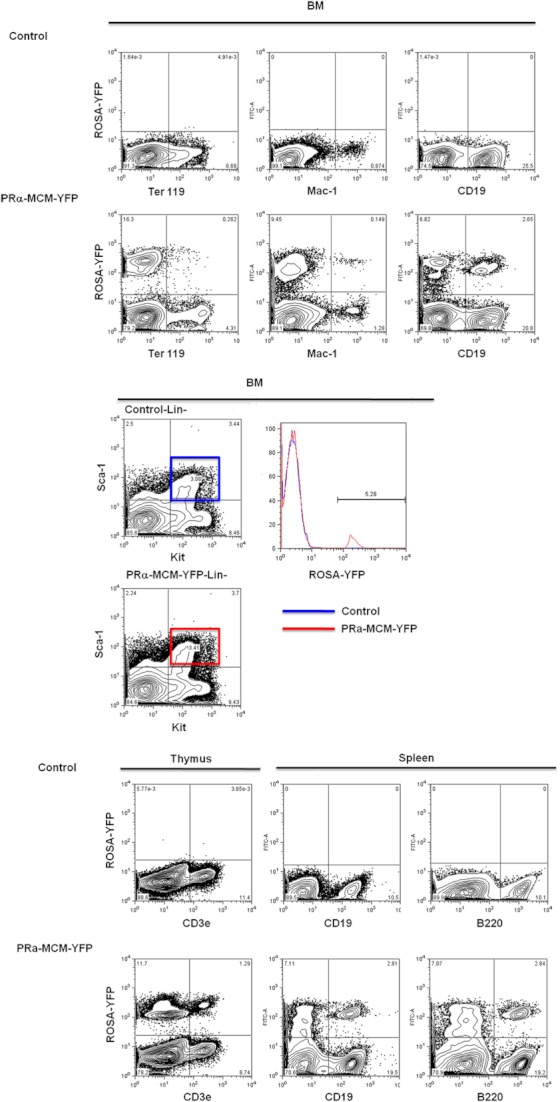
E7.5 PDGFRα+ cells contribute to adult hematopoietic tissue. Bone marrow, thymus, and spleen cells from PRαMCM-YFP adult mice (Tmx injected at E7.5 during pregnancy) were analyzed for the contribution of YFP+-labeled cells. BM contains YFP+ cells in erythroid, macrophage/monocyte, and B cells as well as KSL population. Around 5% of YFP+ cells in KSL population reflects the similar proportion of YFP+ cells in fetal liver KSL population. YFP-labeled cells (E7.5 Tmx) also contribute to B and T cells in spleen and thymus.

**Fig. 5:**
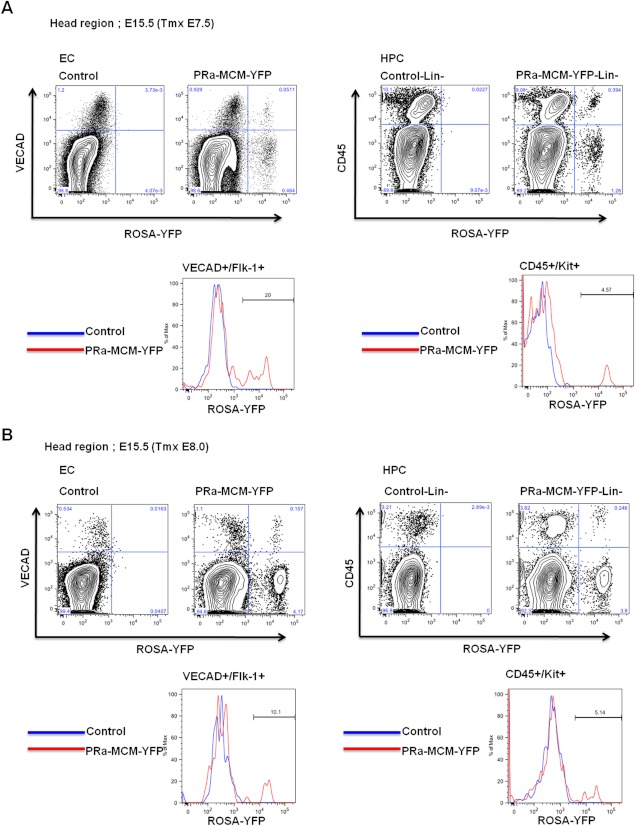
PDGFRα+ cells labeled at E7.5 or E8.0 contribute to cranial ECs. **A:** Head regions from PRα-MCM-YFP embryos were analyzed at E15.5 after Tmx injection into pregnant females at E7.5. By FACS analysis, ∼20% of VE-cadherin+/Flk-1+ cells or ∼4.5% of CD45+/Kit+ cells were YFP+, indicating that E7.5 PDGFRα+ mesoderm contributes to cranial ECs and HPCs. **B:** Pregnant females with PRα-MCM-YFP embryos were Tmx injected at E8.0. Contribution of YFP+ cells to cranial ECs analyzed at E15.5. By FACS analysis, ∼10% of VE-cadherin+/Flk-1+ cells were labeled as YFP+, indicating that a significant proportion of cranial ECs are derived from E8.0 PDGFRα+ mesoderm. Note also that ∼5% of CD45+/Kit+ cells were YFP+, indicating the contribution of E8.0 PDGFRα+ mesoderm into HPCs.

Contribution of embryo proper PDGFRα+ cells into B lymphocytes, reflecting definitive hematopoietic potential, was further confirmed by E8.25 p-Sp explant culture following E7.5 Tmx injection (Fig. 3B). As explant was started from early somite stage before blood circulation, this finding supports that at least part of the definitive hematopoiesis can be established from precursors present in or close to the embryo proper. Fate of YFP-labeled cells by E7.5 Tmx injection was further traced to adult HPCs. In adult PRα-MCM-YFP (Tmx injected at E7.5) mice, we found that labeled cells contributed to multiple lineage BM cells including KSL population as well as lymphocytes in spleen and thymus (Fig. 4). This finding supports the notion that E7.5 PDGFRα+ mesoderm can contribute to hematopoietic progenitors in adult mice. We have observed contribution of PDGFRα+ mesoderm to ECs in PRα-MCM-LacZ embryos at E10.5. Consistent with this finding at E10.5, we confirmed EC contribution also at E15.5 in PRα-MCM-YFP embryos. Around 7 or 10% of VE-cadherin+/Flk-1+ ECs in the cranial region became YFP+ at E15.5 by E7.5 or E8.0 Tmx injection, respectively (Fig. 5A, B), demonstrating the contribution of PDGFRα+ mesoderm into cranial ECs at this stage. As cranial ECs arise from the Etrp/Etv2+ clusters in zebrafish (Proulx et al., 2010) and Etv2 induction is colocalized with Flk-1 (Kataoka et al., 2011), these ECs may be from PDGFRα+/Flk-1+ or PDGFRα+/Flk-1- cells primed to express Flk-1 to acquire vasculogenic potential.

**Fig. 6:**
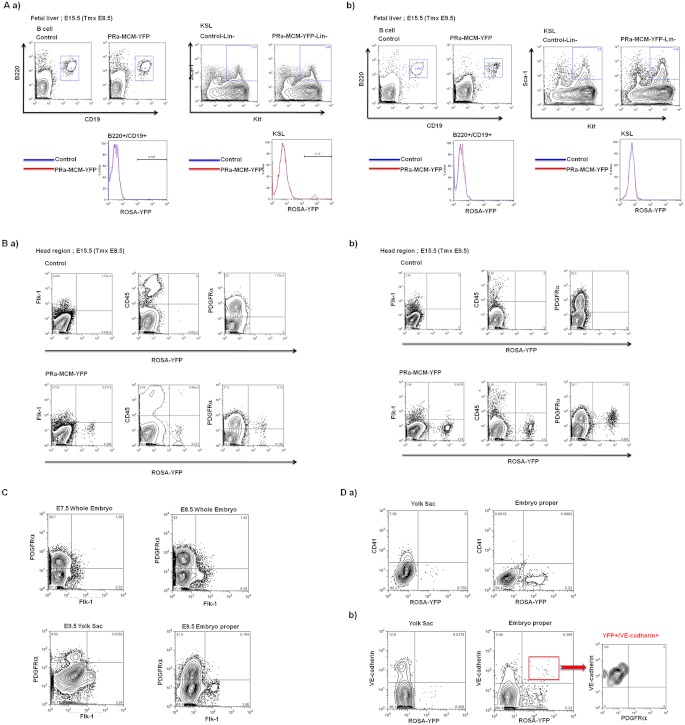
**A**: PDGFRα+ cells labeled at E8.5 contribute to fetal liver HPCs, but with lower efficiency. By E9.5 labeling, almost no PDGFRα+ cells contribute to fetal liver HPCs. **a:** After E8.5 Tmx injection, fetal liver HPCs were analyzed. YFP+-labeled cells contributed to ∼2% of KSL cells but are almost negligible in B cell population, indicating that the contribution of PDGFRα+ mesoderm significantly declines around E8.5. **b:** In fetal liver HPCs, no YFP+ cells were observed after E9.5 Tmx injection into pregnant females. **B:** Contribution of YFP+ cells to cranial ECs/HPCs was analyzed in E15.5 PRα-MCM-YFP embryos after Tmx injection at E8.5 or E9.5 into pregnant females. **a:** In cranial samples, almost no E8.5-labeled YFP+ cells contribute to either Flk-1+ (VE-cadherin+) ECs or CD45+ HPCs, indicating that only early stage PDGFRα+ mesoderm can give rise to cranial ECs and HPCs. Note that the majority of YFP+ cells existed as PDGFRα+ population. **b:** In E15.5 cranial samples, YFP+ cells were present not in Flk-1+ (VE-cadherin+) or CD45+ population, but in the PDGFRα+ population after E9.5 Tmx injection. **C:** PDGFRα+/Flk-1+ cells that exist from E7.5–8.5 decreased profoundly at E9.5. Wild type embryos were stained by anti-PDGFRα and Flk-1 antibodies and analyzed by FACS. Note that the proportion of cells co-expressing PDGFRα and Flk-1 has significantly declined at E9.5 from earlier embryos. **D:** FACS analysis of the PRα-MCM-YFP embryos demonstrating that VE-cadherin+ ECs were labeled within 24 hr after E7.5 Tmx injection. To trace the short-term fate of PDGFRα+ cells, Tmx was injected into pregnant females at E7.5. YS (**a**), and embryo proper (**b**) from PRα-MCM-YFP embryos were analyzed at E8.0 after 12 hr. YFP+ cells were present mainly in the embryo proper (b), but very few in the YS (a). We could not detect any labeled CD41+ primitive HPCs either in yolk sac or embryo proper. Note, however, that part of VE-cadherin+ cells were labeled in the embryo proper (red box), suggesting that VE-cadherin+/PDGFRα-ECs can differentiate from PDGFRα+ mesoderm within 12 hr (b, right panel).

### PDGFRα+ Mesoderm Contribution to ECs and HPCs Is Limited Until E8.5 Embryos

Contribution of PDGFRα+ cells to E15.5 fetal liver HPCs and cranial ECs declined when Tmx was injected at E8.5 (Fig. 6A a, B,a) compared to E7.5–8.0 Tmx injection, becoming almost undetectable by E9.5 injection (Fig. 6A,b, B,b). Since FACS analysis shows that PDGFRα+/Flk-1+ cells profoundly reduced at E9.5 from E7.5–8.5 (Fig. 6C), these findings suggest that PDGFRα+ cells contributing to ECs/HPCs may be mainly from PDGFRα+/Flk-1+ population. This limited time frame for the ECs/HPCs generation from PDGFRα+ cells also suggest that ECs and HPCs develop from PDGFRα+ cells rather immediately while those cells keep Flk-1 expression. To support this idea, we observed the existence of labeled YFP+/VE-cadherin+ cells in E8.0 embryos after E7.5 Tmx injection in 12 hr. It is also likely that these E7.5 PDGFRα+ cells can contribute to hemato-endothelial lineages mainly on the embryo proper side, because we observed labeled cells in PRα-MCM-LacZ embryos mostly in the embryo proper and lateral mesoderm, failing to detect LacZ-labeled cells in the extraembryonic yolk sac region by E7.5 Tmx injection (Fig. 2C, middle and bottom panels). This result was further supported by FACS analysis of PRα-MCM-YFP embryos exposed to Tmx at E7.5. In these embryos, YFP+-labeled cells including YFP+/VE-cadherin+ cells are mainly present in the embryo proper. Absence of YFP+/CD41+ cells also indicates that primitive HPCs in the extraembryonic yolk sac region were not labeled by PRα-MCM transgene in these embryos (Fig. 6D,a, b).

### Functional Significance of PDGFRα+ Mesoderm in Vascular Development and Hematoipoiesis

**Fig. 7:**
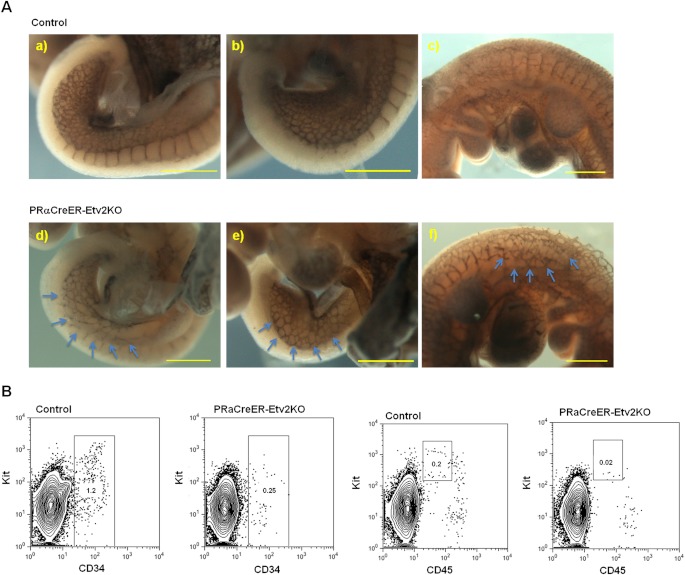
**A:** Etv2 inactivation in early PDGFRα+ mesoderm causes embryonic vascular changes. In PRαBAC-CreER-Etv2KO mice, Etv2 was inactivated in PDGFRα+ mesoderm by Tmx injection at E8.0. PRαBAC-CreER line was used because multi-copy insertions of the BAC construct will provide more efficient Cre activity than PDGFRαMCM one copy knock-in. Control (**a–c**) and PRαBAC-CreEREtv2KO (**d–f**) embryos were exposed to Tmx by injection at E8.0 and analyzed at E9.5 (a, b, d, e) or E10.5 (c, f) by PECAM staining. Note the sparse vitelline vascular network (d,e, arrows) or intersomatic vessels (f, arrows) observed in the PRαBAC-CreEREtv2KO embryos compared with the control. Scale bar=500 μm. **B:** Hematopoietic defects accompanying EC loss in PRαBAC-CreEREtv2KO embryos. Caudal half of E10.5 control and PRαBAC-CreEREtv2KO embryos were analyzed for CD45+ HPCs. Around 50% PRαBAC-CreEREtv2KO embryos showed a reduced number of CD34+ cells and CD45+ HPCs. Reduction in CD45^low^/Kit^high^ population in PRαBAC-CreEREtv2KO embryos suggests the loss of hematopoietic progenitors.

Results presented in the preceding parts indicate that PDGFRα+ cells contribute to ECs and HPCs. However, the functional importance of ECs and HPCs derived from PDGFRα+ mesoderm remains unclear. To examine the functional importance of ECs/HPCs derived from PDGFRα+ mesoderm, we generated PRαBAC-CreER+ Etv2fl/-mice (PRαCreER-Etv2KO) to delete Etv2, which is absolutely required for ECs/HPCs commitment from primitive mesoderm. Etv2 inactivation in PDGFRα+ mesoderm by E8.0 Tmx injection resulted in changes in vitelline plexus or intersomatic vessels (Fig. 7A), indicating the importance of PDGFRα+ mesoderm–derived ECs in vascular development. However, the defects observed were variable among analyzed embryos for several reasons. First, Etv2 expressed in Flk-1+/PDGFRα+ mesoderm may be deleted after Etv2 has activated EC differentiation genes. Due to its transient requirement, PDGFRα+ mesoderm can generate ECs if Etv2 is deleted after EC commitment (Kataoka et al., 2011; Wareing et al., 2012; Lammerts van Bueren and Black, 2012). Second, Etv2 deletion in PDGFRα+ cells can be compensated by other EC sources such as Flk-1+ PDGFRα-population. Nevertheless, the observation that the embryonic vasculature is partly impaired supports the functional significance of ECs derived from PDGFRα+ mesoderm.

**Fig. 8:**
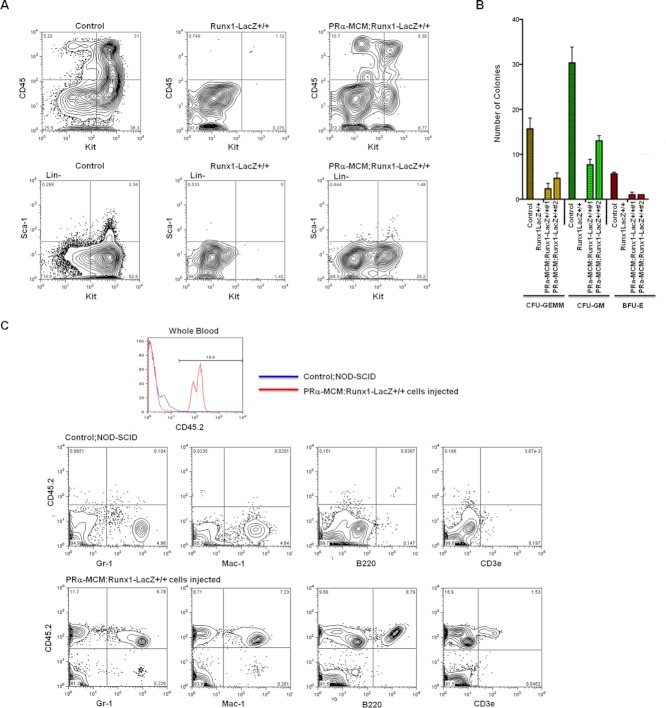
**A**: Restoration of Runx1 expression in PDGFRα+ mesoderm at E7.5 partly rescued. CD45+ or KSL cells in E12.5 fetal liver in Runx1 null background. At E7.5, Tmx was injected into pregnant females having embryos with PRα-MCM transgene over homozygous Runx1-LacZ allele (functionally Runx1 null but Runx1 restorable after Cre recombination). E12.5 fetal liver cells from control, Runx1-LacZ+/+ (functionally Runx1KO), and PRα-MCM;Runx1-LacZ+/+ (functionally Runx1 KO but Runx1 restored after Cre recombination in PDGFRα+ cells) embryos were analyzed by FACS. Note that CD45+ cells were restored substantially in KO embryos carrying PRα-MCM transgene (top panels). A small number of KSL cells were present in PRα-MCM; Runx1-LacZ+/+ embryos but totally absent from Runx1KO embryos (bottom panels). These results suggest that E7.5 PDGFRα+ cells can contribute to HPCs, including hematopoietic stem cells in the fetal liver. **B:** Colony-forming unit assay in PRα-MCM-mediated Runx1 restored fetal liver. Fetal liver cells (10,000 cells/35 mm) were plated in methylcellulose medium from Runx1-LacZ+/-(Control), PRα-MCM;Runx1-LacZ+/+ (PRα-MCM;Runx1KO), and Runx1-LacZ+/+ (Runx1KO) embryos. In PRα-MCM;Runx1-LacZ+/+ embryos, Runx1 expression was restored in E7.5 PDGFRα+ cells by Tmx injection. Note that colony formation was partly recovered in PRα-MCM;Runx1-LacZ+/+ samples compared to Runx1-LacZ+/ +. **C:** Reconstitution of multilineage HPCs by fetal liver cells with Runx1 rescued in PDGFRα+ mesoderm. About 5×10^5^ fetal liver cells from E12.5 PRα-MCM;Runx1-LacZ+/+ (Tmx E7.5) embryos were injected into sublethally irradiated Scid recipient mice. Two months later, contribution of donor-derived cells was assessed by peripheral blood analysis. CD45.2 donor cells were present in the recipient blood and contributed to multiple lineages, including B and T cells, indicating the possible restoration of HSCs from Runx1-restored E7.5 PDGFRα+ mesoderm.

Etv2+ mesoderm has been shown to give rise to both ECs and HPCs. Thus, it is possible that Etv2 ablation in primitive mesoderm can affect HPCs as well. Analysis of caudal half of E10.5 embryo proper revealed reduced CD45+ and CD45^low^/Kit^high^ populations in ∼50% of PRαCreER-Etv2KO embryos compared to control littermates. This HPC defect was seen in embryos showing concomitant reduction of CD34+ cells (Fig. 7B), indicating that HPC defect is accompanying EC loss. Since E10.5 CD45^low^/Kit^high^ population is enriched for hemogenic potential (Nobuhisa et al., 2012), this result indicates that EC loss derived from PDGFRα+ mesoderm can include hemogenic ECs. As another functional test for HPC contribution, fetal liver HPCs were analyzed after Runx1 restoration in E7.5 PDGFRα+ cells in Runx1-LacZ homozygous embryos, which are functionally deficient for Runx1. As reported previously, this Runx1-LacZ allele can restore Runx1 expression after Cre recombination (Samokhvalov et al., 2006; Tanaka et al., 2012). Runx1 restoration by PDGFRα-MCM transgene partly rescued CD45+ or KSL HPCs in PRα-MCM;Runx1-LacZ+/+ fetal liver while these populations were totally absent from Runx1-LacZ+/+ embryos, indicating that there is some rescue by HPCs derived from E7.5 PDGFRα+ cells (Fig. 8A). To further examine the rescue of Runx1 null phenotype in PRα-MCM;Runx1-LacZ+/+ embryos, colony-forming assay was performed using fetal liver cells. While Runx1-LacZ+/+ liver cells failed to generate colonies in methylcellulose, we observed partial recovery of colony-forming units in PRα-MCM;Runx1-LacZ+/+ fetal liver cells (Fig. 8B). It should be noted that Runx1-LacZ+/+ embryos with rescue in PDGFRα+ cells looked pale and modestly anemic and unable to survive beyond E12.5. This is presumably due to the lack of Runx1 rescue in YS, which is reported to be important for primitive and definitive erythropoiesis (Yokomizo et al., 2008). Since PRα-MCM transgene will not induce Cre recombination in YS by E7.5 Tmx injection, erythropoiesis in YS will not be rescued in PRα-MCM;Runx1-LacZ+/+ (E7.5 Tmx) embryos. These results suggest that embryo proper and/or lateral mesoderm Runx1 can restore HPCs to some extent without Runx1 restoration in YS.

To further characterize the rescued cells in the in vivo context, PRα-MCM;Runx1-LacZ+/+ (E7.5 Tmx) fetal liver cells were injected into irradiated recipient mice to evaluate the repopulation capacity. Two months after transplantation into NOD-Scid recipients, we found the significant contribution of PRα-MCM;Runx1-LacZ+/+ (E7.5 Tmx) fetal liver cells into circulating HPCs including monocyte/macrophage, B, and T cells (Fig. 8C). These results suggest that E7.5 PDGFRα+ mesoderm have the potential to give rise to multi-lineage HPCs, possibly HSCs.

### PDGFRα Is Dispensable for the Development of Fetal Liver HPCs

**Fig. 9:**
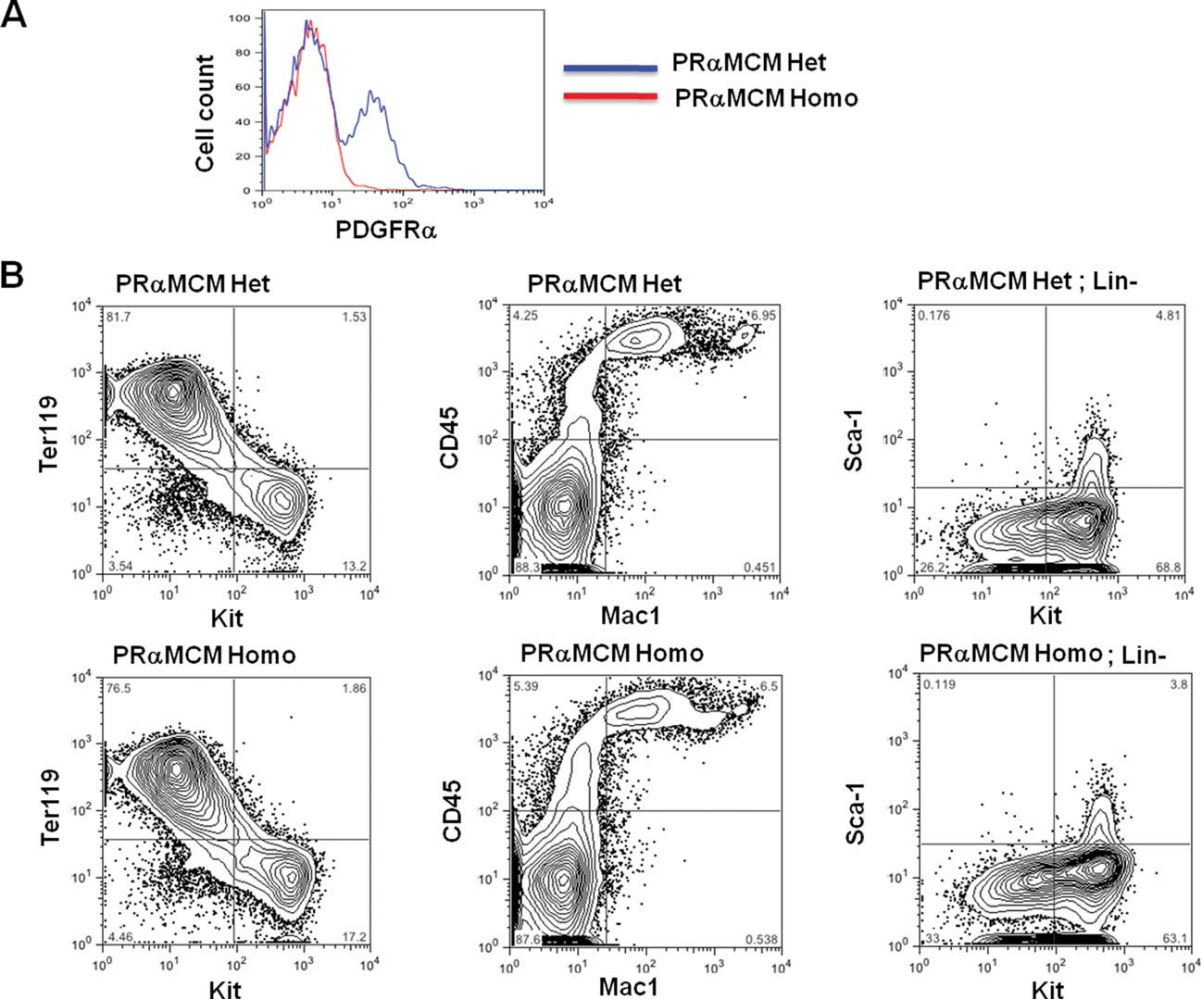
PDGFRα is dispensable for fetal liver HPCs development. PRα-MCM heterozygous mice were used to generate PDGFRα-deficient embryos. Fetal liver HPCs from PRα-MCM heterozygous or homozygous embryos were analyzed at E12.5. **A:** Parts of embryos were stained by anti-PDGFRα antibody confirming that the PRα-MCM homozygous embryo lacked the population expressing PDGFRα **B:** Fetal liver HPCs from PRα-MCM heterozygous or homozygous embryos were analyzed. Homozygous PRα-MCM embryos were assumed to be functionally deficient for PDGFRα, which was confirmed by antibody staining. Ter119+, CD45+, and KSL populations were similar between control heterozygous and PDGFRα deficient embryos, indicating that PDGFRα is dispensable for the fetal liver HPCs development at least until E12.5. Numbers of colony-forming units using heterozygous and homozygous cells were also indistinguishable (data not shown).

Although PDGFRα+ mesoderm was demonstrated to give rise to hemogenic ECs and HPCs, whether PDGFRα signaling is required for hematopoiesis has not been determined. In PDGFRα null embryos, PDGFRα seems to be dispensable for HPCs development since the embryos die apparently from cephalic closure defect or skeletal abnormalities (Soriano, 1997). However, fetal liver seems to be normally formed in null mutants. To confirm that PDGFRα signaling is dispensable for HPC development, FACS analysis of fetal liver cells from homozygous PRα-MCM embryos was performed. PRα-MCM homozygous embryos, demonstrated to be functionally null for PDGFRα, showed indistinguishable HPCs profile from control embryos (Fig.9A, B). This result suggests that PDGFRα itself is dispensable for hematopoiesis to the fetal liver stage. Despite this dispensability in early hematopoiesis, our finding that PDGFRα+ cells give rise to HPCs suggests that PDGFRα is useful for the tracing of particular mesoderm subsets contributing to HPCs.

## DISCUSSION

In this study, we have traced the fate of PDGFRα+ mesoderm cells from early embryos and showed that (1) PDGFRα+ mesoderm in early embryos (possibly, PDGFRα+/Flk-1+ cells) contributes to ECs and HPCs including B lymphocytes and KSL cells, (2) PDGFRα+ mesoderm at E7.5–8.0 giving rise to ECs and HPCs represents a distinct population from extraembryonic Runx1+ population, and that (3) ECs and HPCs from PDGFRα+ mesoderm have functional significance revealed by Etv2 deletion or Runx1 restoration, respectively. It has been demonstrated that all HPCs and ECs are derived from Flk-1+ cells in mice.

PDGFRα is another early mesoderm marker mainly for paraxial mesoderm giving rise to muscle or mesenchymal cells. In both embryos and differentiated ES cells, PDGFRα+/Flk-1+ cells are present as primitive mesoderm that differentiate into cardiomyocytes, skeletal muscle, mesenchymal cells, and hemato-endothelial lineages. We showed by cell sorting and reculture experiments that PDGFRα+/Flk-1- or PDGFRα+/Flk-1+ cells generate ECs and HPCs in ES differentiation culture. By time point–specific labeling in early mouse embryos, we demonstrated that ECs and HPCs derived from PDGFRα+ mesoderm exist as a physiological population in developing embryos and postnatal mice. PDGFRα+ mesoderm first emerge at E6.5–E7.0 around the proximal part of the primitive streak (Kataoka et al., 1997). Thus, Tmx injection at E6.5 labeled cells that migrate to blood islands corresponding to the proximal part of the extraembryonic mesoderm. As PDGFRα+ cells exist mostly on the embryo proper side at E7.5, Tmx injection at E7.5 labeled cells giving rise to embryo proper structures including somites, head mesenchyme, and dorsal aorta that hardly overlap with those labeled by E6.5 injection (Fig. 2C). This result demonstrates that at least in these developmental stages Tmx activity does not persist beyond 24 hr (Zovein et al., 2008) and validates the time frame specificity of the PRαMCM transgene. This difference of labeling along the primitive streak is also consistent with the injected-dye tracing studies that almost distinguished the descendants migrating into extra or intra embryonic regions from spatiotemporally different parts of the primitive streak (Wilson and Beddington, 1996; Kinder et al., 1999).

At E7.5–8.0, immunostaining revealed that PDGFRα+ mesoderm population exists in embryo proper and lateral mesoderm close to the embryo proper side, which is distinct from Runx1+ population in the extraembryonic yolk sac. Several places in early embryo are proposed as candidate sites generating HSCs, including yolk sac, p-Sp, intraembryonic caudal mesenchyme, allantois, and placenta. Our finding that PDGFRα+ cells labeled at E7.5 contribute to ECs and HPCs including B lymphocytes and fetal liver and BM KSL cells suggests that at least part of the definitive hematopoietic cells originate in locations corresponding to p-Sp including part of the lateral mesoderm or allantois distinct from extraembryonic YS. This notion was also supported by p-Sp culture experiment from PRα-MCM-YFP embryos exposed to Tmx at E7.5. Furthermore, we showed that PDGFRα+ mesoderm labeled at E7.5 can give rise to multi-lineage adult BM HPCs. Repopulation of Runx1 restored cells derived from E7.5 PDGFRα+ mesoderm also supports that PDGFRα+ cells can be a source for HSCs.

It is noteworthy that p-Sp region contains PDGFRα+/Flk-1+ cells that are likely to be the source for ECs and HPCs. Previous reports including ours suggest that Flk-1 (and Etv2 co-expressed in Flk-1+ population) is necessary for EC/HPC development in mouse embryos. Whether PDGFRα+ mesoderm–derived ECs/HPCs are generated directly from PDGFRα+/Flk-1+ population or there are PDGFRα+/Flk-1- cells primed to express Flk-1 needs to be determined.

We also noted that PDGFRα+ cells contribute to the ventral side of the aorta where hemogenic ECs are present. In short-term tracing experiment, Tmx injection at E7.5 labeled cells that were distributed in the caudal-lateral mesoderm at E8.0 (Fig. 2C, middle panels). Although the molecular mechanism as to how labeled cells first distribute laterally before settling in the paraxial region at E8.5 (Fig. 2C, bottom panels) is unclear, mesoderm cells in this region at E8.0 overlap cells labeled by Hoxb6Cre transgene, which is known to label cells that contribute the hemogenic ECs on the ventral side of the aorta (Wasteson et al., 2008). Indeed, descendants of Hoxb6Cre-labeled cells have been demonstrated to contribute to adult bone marrow HPCs (Zovein et al., 2010), supporting our result that part of early PDGFRα+ cells can contribute to KSL population in fetal liver and adult HPCs. In chicken, it is postulated that ECs derived from splanchnopleura with hemogenic potential distribute on the ventral side of aorta, which are replaced by non-hemogenic somite-derived ECs (Pouget et al., 2006). In mouse, PDGFRα+ cells are assumed to give rise mainly to paraxial mesoderm including somites. However, we found that early PDGFRα+ mesoderm contributes to hemogenic ECs possibly through lateral mesoderm, which corresponds to the Hoxb6-positive area. The molecular mechanism as to how part of the PDGFRα+ cells can give rise to lateral mesoderm in addition to somites is unknown at present. Nevertheless, we should note that at E7.5–E8.0 VEGF is broadly expressed in the visceral endoderm over extraembryonic-lateral mesoderm (Miquerol et al., 1999), suggesting that PDGFRα+ cells responding to VEGF expressed in that area obtain the hemato-endothelial fate.

This study also showed the ECs and HPCs derived from PDGFRα+ mesoderm are functionally important in vascular development and hematopoiesis by genetic manipulation of key factors in PDGFRα+ mesoderm. Previous investigation showed that ECs can be derived from early somites where PDGFRα is highly expressed. Finding that Etv2 deletion in PDGFRα+ mesoderm causes vascular defect also suggests that part of the paraxial mesoderm may be a significant source of ECs for embryonic vascular development. Despite the defective vascular patterning in vitelline or intersomatic vessels, we were unable to detect significant changes in the anterior part of the vasculature including cranial region of PRαCreER-Etv2KO embryos. It has been reported that anterior part of the mouse embryonic vasculature forms earlier than the posterior part (Drake and Fleming, 2000). Thus, we speculate that compared to the posterior region, more ECs in the anterior part already might have passed the phase of transient Etv2 requirement at E8.0 when Tmx was injected. Additionally, the finding that PDGFRα+ cells hardly contribute to HPCs or ECs if Tmx was injected after E9.0–9.5 suggests that the vascular phenotype may be coming from Etv2 deletion in PDGFRα+ cells until E8.5. It is also noteworthy that caudal-lateral mesoderm, labeled by Hoxb6 Cre, that gives rise to vitelline plexus overlaps PDGFRα+ area, which is consistent with the change we observed in vitelline vasculature in PRαCreER-Etv2KO embryos.

It has been controversial whether HSCs are mainly coming from extraembryonic yolk sac or intraembryonic part. We could observe some restoration of HPCs in Runx1-deficient fetal liver by Runx1 restoration in PDGFRα+ early mesoderm. PRα-MCM;Runx1-LacZ+/+ (E7.5 Tmx) embryos were apparently pale, partly due to the bleeding as seen in Runx1 null embryos. Runx1 may be dispensable for primitive erythropoiesis but required for the proper erythroid development, making Runx1 null embryos modestly anemic (Yokomizo et al., 2008). Based on the labeling experiments, we can predict that activation of PRαMCM transgene at E7.5 will fail to rescue Runx1 in extraembryonic YS blood island. Runx1 deficiency in YS blood island will create modest defects in erythropoiesis in PRαMCM; Runx1-LacZ+/+ embryos even after E7.5 Tmx injection. However, some rescue in Runx1 dependent CD45+ and KSL populations, colony-forming units, and transplantable cells in PRα-MCM;Runx1-LacZ+/+ fetal liver after E7.5 Tmx injection demonstrate that embryo proper side mesoderm has the potential to contribute to hemogenic ECs and possibly HSCs independently of YS extraembryonic mesoderm. So far, the origin of HSCs in early mouse embryos has been mainly investigated by explant culture of specific parts without any cell tracing from specific areas from early embryogenesis. Runx1-MCM-based cell tracing was used to follow the E7.5 extraembryonic yolk sac mesoderm. PRα-MCM transgene gave us an opportunity to perform labeling or gene manipulation specifically on the embryo proper side including lateral mesoderm to address where HPCs can originate. Indeed, PDGFRα+ cells labeled at E7.5 distribute exclusively on the embryo proper side at E8.0–8.5, indicating that labeling (Cre activity) is limited to the embryo proper and lateral mesoderm close to embryo proper. HPC rescue to achieve repopulating fetal liver cells by Runx1 restoration in E7.5 PDGFRα+ cells suggests that these cells can be the origin of HPCs and HSCs. On the other hand, several reports claim that extraembryonic YS can be a source for HSCs (Yoder et al., 1997; Tanaka et al., 2012). How YS-derived HPCs/HSCs co-exist with PDGFRα+ mesoderm–derived HPCs or how they interact needs further investigation.

We have shown that PDGFRα+ mesoderm generates ECs/HPCs in embryos as well as in ES differentiation culture. While Flk-1 has been used as a primary marker to identify HPC precursors in ES differentiation, recent reports suggest that Flk-1+ cells are heterogenous. Irion et al. (2010) reported that two distinct Flk-1+ populations with different hematopoietic potential are generated from differentiated ES cells, showing that the later day-5 Flk-1+ cells have more definitive hematopoietic potential than day-3 population. It is notable that E7.5–8.0 Flk-1+/PDGFRα+ cells are present in lateral mesoderm, which is labeled by Hoxb6 Cre transgenic line and shown to become hemogenic ECs. This raises the possibility that introducing PDGFRα may be useful for marking “hemogenic” mesoderm if used at appropriate time points during differentiation. In human ES differentiation, CD34+ cells exist as PDGFRα+ (Davis et al., 2008) supporting the usefulness of PDGFRα to monitor HPC differentiation from ES cells. Some PRαCreER-Etv2KO embryos showed vascular patterning defect in vitelline plexus that is tightly linked to definitive HSC development (Zovein et al., 2010). These results together support the idea that PDGFRα may serve as an additional surface marker to subdivide Flk-1+ cells and enrich useful populations if used in appropriate ES differentiation stages in HPC induction.

## EXPERIMENTAL PROCEDURES

### Mice

PRαMCM mice were generated by knocking Tmx-inducible MCM cDNA (gift from M. Reth) into the PDGFRα locus. Adenoviral splice acceptor sequence (SA) was added to 5′ side of the MCM cDNA to prevent cryptic splicing. Homology arms used for targeting were 5′ side, 79,307–85,194; 3′ side, 85,253–89,284 from RP23-–55P22. Frt sites flanked Neo cassette was placed 3′ to the SA-MCM-polyA sequence. The targeting vector constructed was linearized by AscI and electroporated into TT2 ES cells to obtain targeted ES cells, which was used to generate mice by standard procedure. MerCreMer was activated in embryos by injecting Tmx into pregnant females (approximately 100 μg/g [body weight] 4-OH tamoxifen [Tmx]; Sigma, St. Louis, MO, H7904). Detail for the Etv2 conditional allele will be reported elsewhere. In brief, two loxP sites were placed in the Etv2 locus to flank the DNA-binding domain. Hetrozygosity of the DNA-binding domain deletion caused no obvious phenotype while homozygosity recapitulated the Etv2 null phenotype as reported. PDGFRαBAC-CreER (PRαBAC-CreER) line was from B. Richardson (University College London) (Rivers et al., 2008). Runx1-IRES-GFP knock-in mice were a gift from Dr. Lorsbach (St. Jude Children's Research Hospital, Memphis, TN) (Lorsbach et al., 2004). For Runx1 recue experiments, mice were crossed to get PRα-MCM;Runx1-LacZ+/+ alleles that harbor PRα MCM transgene over restorable Runx1 null allele. For colony-forming assays, 10,000 fetal liver cells/35 mm dish were plated in methylcellulose medium (Stem Cell Technology, Vancouver, Canada, M3434). Sublethally radiated (249.9cGy) NOD-Scid mice (Charles River, Wilmington, MA) received a half million fetal liver cells from PRα-MCM;Runx1-LacZ+/+ embryos. After 2 months, peripheral blood samples were analyzed by indicated antibodies to evaluate the contribution of engrafted cells.

### Tissue Culture

Explant culture to generate B cells and mice for Runx1 restoration and labeling have been described (Tanaka et al., 2012). In brief, the caudal part of embryo proper was separated from E8.25 early stage embryos and explanted on OP9 cells in the presence of IL-7 (10 ng/ml) and Flt-3L (10 ng/ml). After 2 days, explant was dissociated and continued to be cultured for an additional 12–14 days in the same condition. Wells positive for growing HPCs were analyzed by FACS using indicated antibodies.

### Immunostaining, FACS, and LacZ Staining

Whole mount immunostaining was performed as described. Primary antibodies used for each antigen was as follows: Flk-1 (goat polyclonal [R&D, Minneapolis, MN], or rat monoclonal [e-Bioscience, San Diego, CA]), PDGFRα rabbit monoclonal; CST, goat polyclonal [R&D], or rat monoclonal [APA5], e-Bioscience), Runx1 (EPR 3099; epitomics 2593-1), CD31(MEC 13.3, BD). For HRP staining, appropriate secondary antibodies (goat anti-rat-HRP (KPL), goat ant-rabbit-HRP, and Donkey anti-goat HRP (Jackson Immunoresearch, West Grove, PA) were used at a dilution of 1/800. For fluorescent staining, Alexa488, Alexa546 (Invitrogen, Carlsbad, CA), and Cy5 (Jackson Immunoresearch) were used at a dilution of 1/1,000. All FACS antibodies were from Biolegend (San Diego, CA). Fetal liver cells were recovered by smashing the embryonic liver through cell strainer, incubated in Fc block antibody, and then stained by indicated antibodies. For head part analysis, tissue was minced into small pieces, dissociated by trypsin and 18G needle (after trypsin neutralization), recovered in serum containing medium for 15 min, incubated in Fc block antibody, and then stain by indicated antibodies. LacZ staining was performed after fixing embryos in 2% paraformaldehyde/2% glutaraldehyde for 1 hr, washed twice by PBS and then incubated in X-Gal (1 mg/ml) containing solution.
